# Can Peer Review Be Kinder? Supportive Peer Review: A Re-Commitment to Kindness and a Call to Action

**DOI:** 10.1177/20543581221080327

**Published:** 2022-05-01

**Authors:** Catherine M. Clase, Elizabeth Dicks, Rachel Holden, Manish M. Sood, Adeera Levin, Kamyar Kalantar-Zadeh, Linda W Moore, Susan J. Bartlett, Aminu K. Bello, Clara Bohm, Darren Bridgewater, Josee Bouchard, Dylan Burger, Juan Jesús Carrero, Maoliosa Donald, Meghan Elliott, Maya J. Goldenberg, Meg Jardine, Ngan N. Lam, W. Joy Maddigan, François Madore, Thomas A. Mavrakanas, Amber O. Molnar, G. V. Ramesh Prasad, Claudio Rigatto, Karthik K. Tennankore, Elena Torban, Laurel Trainor, Christine A. White, Sunny Hartwig

**Affiliations:** 1 Department of Medicine, McMaster University, Hamilton, ON, Canada; 2 Department of Health Research Methods, Evidence, and Impact, McMaster University, Hamilton, ON, Canada; 3 Department of Medicine, St Joseph’s Healthcare Hamilton, ON, Canada; 4 Canadian Journal of Kidney Health and Disease, Canada; 5 Department of Medicine, Queen’s University, Kingston, ON, Canada; 6 Department of Medicine, The Ottawa Hospital Research Institute, The Ottawa Hospital, ON, Canada; 7 Department of Medicine, The University of British Columbia, Vancouver, Canada; 8 University of California, Irvine, Orange, CA, USA; 9 Houston Methodist Hospital Department of Surgery, Houston, TX, USA; 10 Department of Medicine, McGill University, Montreal, QC, Canada; 11 Division of Nephrology, Department of Medicine, University of Alberta, Edmonton, Canada; 12 Department of Internal Medicine, Chronic Disease Innovation Centre, University of Manitoba, Winnipeg, Canada; 13 Department of Pathology and Molecular Medicine, McMaster University, Hamilton, ON, Canada; 14 Department of Medicine, Université de Montréal, QC, Canada; 15 Kidney Research Centre, Ottawa Hospital Research Institute, University of Ottawa, ON, Canada; 16 Department of Medical Epidemiology and Biostatistics, Karolinska Institutet, Stockholm, Sweden; 17 Cumming School of Medicine, University of Calgary, AB, Canada; 18 Department of Philosophy, University of Guelph, ON, Canada; 19 National Health and Medical Research Council Clinical Trials Centre, University of Sydney, NSW, Australia; 20 Division of Nephrology, Cumming School of Medicine, University of Calgary, AB, Canada; 21 Department of Community Health Sciences, University of Calgary, AB, Canada; 22 Faculty of Nursing, Memorial University of Newfoundland, St. John’s, Canada; 23 Division of Nephrology, Department of Medicine, University of Toronto, ON, Canada; 24 Department of Medicine, University of Manitoba, Winnipeg, Canada; 25 Department of Medicine, Dalhousie University, Halifax, NS, Canada; 26 Nova Scotia Health Authority, Halifax, Canada; 27 Department of Psychology, Neuroscience & Behaviour, McMaster Institute for Music and the Mind, McMaster University, Hamilton, ON, Canada; 28 University of Prince Edward Island, Charlottetown, Canada

**Keywords:** humility, kindness, peer review, supportive review, truth

## Abstract

Peer review aims to select articles for publication and to improve articles before publication. We believe that this process can be infused by kindness without losing rigor. In 2014, the founding editorial team of the *Canadian Journal of Kidney Health and Disease* (CJKHD) made an explicit commitment to treat authors as we would wish to be treated ourselves. This broader group of authors reaffirms this principle, for which we suggest the terminology “supportive review.”

## Abrege

L’évaluation par les pairs vise à sélectionner les articles à publier et à en améliorer le contenu avant publication. Nous sommes d’avis que ce processus peut être fait avec bienveillance sans perdre en rigueur. En 2014, l’équipe de rédaction fondatrice du *Canadian Journal of Kidney Health and Disease* (CJKHD) a pris l’engagement ferme de traiter les auteurs comme ses membres souhaiteraient eux-mêmes être traités. Aujourd’hui, notre groupe élargi d’auteur(e)s réaffirme ce principe pour lequel nous proposons la terminologie « évaluation constructive ».

When the *Canadian Journal of Kidney Health and Disease* (CJKHD) was launched in 2014, the editors outlined 9 points where we hoped the journal could make a unique contribution.^1^ These 9 points were later expanded to eleven, and then to seventeen.^2^ A fundamental principle from the beginning was the concept of supportive review:

Our explicit and unique policy is to address our authors as our colleagues with the aim to improve submitted works, regardless of our decision to publish. We will be no less scientifically and constructively critical, but we will write our reviews as we would wish to be written to ourselves.^1^

Seven years later, this principle continues to be a core value. Although known to be beset with problems, peer review has been called the “least worst” system available to assess articles for publication.^3^ In open-access publishing, CJKHD editors and others have attempted to avoid publication bias by explicitly publishing work that is internally valid, with less consideration given to its perceived importance, impact, or the direction of the findings. The *Canadian Journal of Kidney Health and Disease* (CJKHD) attempts to minimize racism^4-6^ and gender bias^6-10^ through explicit commitments to equity, diversity, and inclusion in all our activities, not least being the maintenance of an editorial team and editorial board that includes a high proportion of women and men from various ethnicities. As it happens, the 2 editors-in-chief (founding and current) are both women, though both are white. We minimize error detection problems by choosing trained, detail-oriented reviewers, often early and mid-career, and by admitting research trainees enrolled in the interdisciplinary Kidney Research Scientist Core Education and National Training (KRESCENT) program to the editorial board.^8,11^

Peer review can lack constructive positive feedback and be downright unkind. Little has been written on this. The diverse group of editors at CJKHD spanning early- to senior-career status identified this problem from our own anecdotal experiences with peer review. Publications on the experience of peer review are limited. Editors in Africa identified negative interpretations of reviews from international journals as a factor leading to African scientists publishing in predatory journals,^12^ a phenomenon which has also been anecdotally observed for submissions in which most authors were based in China. In a poll of editors of Nature Research journals, 23% of respondents had encountered examples of inappropriate language in the peer-review process; among authors, 5% said that their experience with peer review were broadly negative and 47% that they were neither negative or positive.^13^ In an opinion piece giving advice to young scientists, Kathleen Roe wrote about her first experience of peer review: “In language that was unfamiliar to me at the time, the editor spoke of “suitability” and “journal standards,” and then referred me to 4 pages of single-spaced, sharply worded, and low-context critique by 2 peer reviewers.”^14^ From the perspective of authors, who must work with whatever they receive from journals, her cognitive-behavioral approach to developing resilience is likely quite helpful: “They may be abrupt, harsh, even unkind in their word choices. That’s on them—it’s just a peer review.”^14^ However, from the perspective of editors for *CJKHD*, we felt compelled to do better and to avoid abusive processes.

Concrete examples of “helpful” and “unhelpful” feedback are widely available and endorsed by journals,^15,16^ and the Committee on Publication Ethics explicitly speaks of fairness, requiring reviewers to “refrain from making unfair negative comments or including unjustified criticisms of any competitors’ work that is mentioned in the manuscript.”^17^ Other suggestions to make reading and responding to review easier for authors include not attempting to rewrite the manuscript according to the reviewer’s own style or preferences and writing “constructively.”^15,17^ The use of quality checklists^18,19^ as fair external standards would be expected to improve the transparency and objectivity of the review. Open review, in which reviews are signed by the reviewer, would tend to improve transparency and accountability but has not been shown to improve the quality of reviews,^20^ and we have therefore chosen not to implement this. Reviewers suggested by the author might be more likely to write constructively; however, a system in which authors suggest reviewers is open to fraud and has been an underlying factor in retractions.^21,22^ In our view, none of these ideas goes as far as we wish to go in explicitly requiring kindness and respect.

Constructive kindness requires empathy (“write as we would wish to be written to ourselves”),^1^ and in our training sessions, we further operationalize this by suggesting reviewer and editors write “as if to your treasured mentor or to your most junior trainee.” Kindness is also a good in itself, a prosocial virtue, related to a desire to help others effectively and to altruism.^23^ Reviewing is inherently an altruistic act, motivated by a desire to improve scientific knowledge and to give back to the community.^24^ Anecdotally, and from our personal experiences as authors, though we and others very much appreciate kind words and other recognitions from editors, the recognition that peer-reviewing receives in academic systems is so weak that we do not consider it a reward. In contrast, egotistical motivations^23^ for reviewing include perpetuation of one’s own mindset, the status quo, current practices, current doctrines, and interpretation of evidence; a paradoxical sense of superiority in being asked to provide peer review (i.e., review of an equal); and satisfaction from the power imbalance between reviewer and reviewed.

We created the term “supportive review” to describe our idea, believing it to be novel to spell out constructive kindness in this way. A Medline search from 1946 to 2021, conducted 2021-06-06 using the keywords “peer review” and synonyms for supportiveness and kindness identified 194 articles, 5 of which mentioned this issue. Advice to authors from one editor explicitly suggested to “be kind” in the opening paragraph, but then to move to “criticism” as if these are mutually exclusive concepts.^25^ Since 1998, the Journal of Genetic Counseling has had an explicit policy of mentoring early-career researchers, operationalized through an “encouraging” philosophy and the ability of new authors to consult with the editor-in-chief about their manuscripts.^26^ A letter to the *BMJ* in 2002 called on statistical reviewers to be “thoughtful, constructive, and encouraging.”^27^

Writing in 2006, Dutta suggested 10 commandments for reviewers, many of which are in the spirit of supportive review (e.g., “approach reviewing as a collaborative task,” “put aside your ego,” and “be reflexive”) (Table 1).^28^ Commandment 9 is specifically “Encourage!” urging the reviewer to “think of reviewing as a positive opportunity for touching the life of another scholar.” Commandment 10, “do unto others as you would have them do unto you,” specifically aligns with our own “write as we would wish to be written to ourselves.” A 2010 qualitative analysis of 99 reviews of rejected manuscripts found that reviewers “made significant efforts at kindness and gentility” and recognized that the importance of simultaneously maintaining a supportive and respectful community while entertaining rigorous and interesting debate is common to other aspects of academic life, for example, seminars and academic rounds.^29^ Ploegh, writing in a Nature op ed in 2011, highlighted the power imbalance between reviewer and authors, and wastefulness and delays associated with reviewers’ demands for additional experiments that are not essential to the work itself, sometimes called “reviewer experiments,” recommending that reviewers decide on the merits of the manuscript as submitted, and that editors exercise judgement and provide direction to authors when reviewers suggest further experiments.^30^ A Nature editorial in 2020 documenting issues with peer review was entitled “*Peer review should be an honest, but collegial, conversation*”^13^ and a career column by Clements in the same journal called for less meanness and unprofessionalism and more “positive comments” in scientific reviewing.^31^ Clements also suggests formal reviewer training as part of the way forward.^31^ A 2021 scoping review found that 58% of instructions to reviewers included some comment on “how to behave towards authors or how to keep. . .positive and constructive tone.”^32^

**Table 1.** The 10 Commandments of Reviewing: The Promise of a Kinder, Gentler Discipline by Mohan J Dutta,^28^ Paraphrased for Clarity as a Stand-Alone List, and Aligned With Indigenous 7 Teachings Principles as Endorsed in the Aims and Scope of the *Canadian Journal of Kidney Health and Disease*.

	Suggestion	Seven teachings principles
1	Approach reviewing as a collaborative task	Respect
2	Put aside your ego	Humility
3	Be reflexive	Respect
4	Understand the paradigms of the research discipline or methodology in question	Wisdom
5	Understand the limitations of the project	Wisdom, honesty, humility, and truth
6	Do not feel that you need to demonstrate how much you know	Wisdom and humility
7	Be specific in your recommendations	Wisdom, bravery, respect, honesty, and truth
8	Provide feedback in a timely manner	Respect
9	Encourage!	Love and humility
10	Do unto others as you would have them do unto you	Respect and love

We later learned that the term “supportive peer review” was used in 2008 to describe the process of mutual review of higher education institutions through the European University Association’s Institutional Evaluation Program.^33^ There is, however, a very limited literature on supportive review in peer review: we were unable to identify previous publications focussed on this topic and are not aware of other journals that specifically endorse kindness.

*Canadian Journal of Kidney Health and Disease* operationalizes our professional, supportive review policy through reviewer selection, choosing scientists whom we know to be rigorous and believe will provide supportive reviews. Recruitment of associate editors is purposive, and the process of recruitment explicitly includes discussion of the importance of supportiveness as one of the journal’s guiding principles. All associate editors are trained in supportive review. Editors are permitted to remove statements from reviewer’s reports before transmitting them to authors: most editors recognize the necessity for this but few journals have an explicit policy.^34^ (Reviewers are copied on both reviews and editorial comments as they go out to authors, but we do not specifically notify them if we delete a statement.) We provide editorial comments above the peer reviews, in which we attempt to help authors understand what we consider most important, and where we rephrase kindly as suggestions important concepts that might be more bluntly stated in the review below. We are concerned about the power imbalance between reviewer and author and attempt to redress this imbalance by giving direction. For example, if a reviewer suggests a radically different analytic approach from that taken originally, or large-scale supplementary analyses, or additional experiments that are not critical to the interpretation of the existing work, we exercise editorial judgment and indicate to authors whether we regard these as essential changes to the manuscript, or whether we invite the authors’ perspective and their constructive response to the suggestion. In Canada, many trainees at the postdoctoral and new investigator level, who are working in kidney health and disease, are enrolled in the competitive Kidney Research Scientist Core Education and National Training program (KRESCENT). All trainee scientists are trainee reviewers for *CJKHD*. Curriculum developed by journal editors for trainee reviewers in the KRESCENT program includes the importance of kindness, concrete ideas about how to write using judgment, wisdom, professionalism, and supportiveness, and feedback on their own reviewing practices assessed on real manuscripts (Table 2). In this way, we are also building capacity for future peer review in kidney science. We have the most control and the highest standards in the feedback written by associate editors and deputy editors, who provide their own comments in addition to at least 2 peer reviews. We have learned to recognize the importance of not losing clarity through kindness^29^—in the first year of our work, there was one occasion where an author did not realize that the final decision was rejection because of the way the letter was worded. Finally, we are aware of the “perils of praise”^35,36^ and remain focussed on the work, using objective language to describe what is good in the same way that we do when we have suggestions for improvement.

**Table 2.** The Kidney Research Scientist Core Education and National Training Program (KRESCENT) Holds an Annual Workshop on Peer Review: CJKHD Editors Provide Curriculum as Shown. (Slide Set Available on Request.)

1. Confidentiality
2. Known problems with peer review, a literature review
a. Publication bias
b. Other biases, particularly those relating to equity, diversity, and inclusion
c. Error detection
d. Disagreement between reviewers by chance
3. Ideas for improvement
a. Improved instructions
b. Structured articles and abstracts
c. Checklists such as those curated by the EQUATOR network^20^
d. Selection and deselection of reviewers
e. Feedback to reviewers
f. Professional colleges of reviewers
g. Write kindly
4. Feedback on reviews prepared by trainees in pairs on submitted manuscripts

*Note.* CJKHD = *Canadian Journal of Kidney Health and Disease*.

Our workflow also supports our processes. A deputy editor reviews each submitted manuscript to decide if it should be peer reviewed. All decisions to reject without review are discussed, usually within a few days, by all deputy editors (there are currently 3) and the editor-in-chief. Decisions to accept peer review that is submitted with the work, in keeping with our portable review policy, are made by this same group, and an associate editor. This process takes into account the quality of the review, the quality of the response to the review, and the source of the peer review (as a surrogate because we do not know who the anonymous peer reviewers are, in this context). For manuscripts that undergo peer review, once peer review has returned, the associate editor writes editorial feedback to augment the reviews, show emphasis, and suggest solutions. The associate editor’s draft email and decision are reviewed by one of the deputy editors before being sent. The editor-in-chief reviews all emails to authors. Each manuscript is considered on its own merits. There are no editorial meetings in which the relative claims of different papers compete. Cross-checking and review of each others’ work in this way maintains the principles established in editor training.

Editors of the Journal of Renal Nutrition express their support for the ideas represented here by co-authorship of this editorial. Editors of the American Journal of Physiology, Renal Physiology; AJKD; CJASN; Clinical Nephrology; JASN; Kidney International; Nature Reviews Nephrology; NDT; Nephron; Pediatric Nephrology; and Renal Society of Australia Journal were also supportive. Nature Reviews Nephrology, NDT, and Pediatric Nephrology will also respond in detail through the pages of their own journals. Editors in any discipline interested in joining a working group to develop and promote supportive review should contact the corresponding author.^37,38^

Confidence in science is diminished when scientists insist on a perspective despite evidence to the contrary, or when they engage in dubious statistical practices. Our team works with humility to recognize and avoid publishing judgments that are biased or science that is not internally valid. Getting peer review right is a key aspect of maintaining research quality and improving public trust.^39^ We ask other journals to consider a commitment to gradual implementation of supportive review, recognizing that some disciplines are likely better prepared for this than others. We foresee growing as a community of practice and recognize, as researchers, reviewers, editors, and authors, that we are one body sharing kinship in the critical evaluation of each others’ work in the pursuit of advancing knowledge (Tables 1 and 2).
